# Tetrad of Narcolepsy Type 1: Treatment and Management

**DOI:** 10.7759/cureus.55331

**Published:** 2024-03-01

**Authors:** Kanishka Gandhi, Suraiya Ferdous

**Affiliations:** 1 Physiology, Jawaharlal Nehru Medical College, Datta Meghe Institute of Higher Education & Research, Wardha, IND; 2 Physiology, Jawaharlal Nehru Medical College, Datta Meghe Institute of Medical Sciences, Wardha, IND

**Keywords:** orexin, hypocretin, rapid eye movement, daytime sleepiness, hallucinations, delayed diagnosis, sleep disorders, symptoms, catalepsy, narcolepsy

## Abstract

Narcolepsy is a chronic condition that brings about excessive daytime sleepiness. It can be classified into two types: narcolepsy type 1 (presence of cataplexy, which is marked by weakness of muscles) and narcolepsy type 2 (without cataplexy). It is generally underdiagnosed, which results in delayed diagnosis of the condition. It has more prevalence in the United States of America as compared to India. The narcoleptic tetrad consists of excessive daytime sleepiness (EDS), cataplexy, sleep paralysis, and hypnagogic hallucinations. Rapid eye movement (REM) sleep behavior disorder is another characteristic feature. Research about narcolepsy has been carried out for about 145-150 years, but it is only in the last 18-20 years that there has been advancement in the underlying pathophysiology, diagnosis, and, thus, availability of better treatment. Both pharmacological and non-pharmacological methods are preferred in treating narcolepsy, yet there is no cure for it. Since the knowledge regarding this condition is very limited, it is often misunderstood, and dealing with it is mentally and socially draining, often causing anxiety in the patients, feeling of social isolation, and other significant impacts on the quality of living. Raising awareness about narcolepsy is vital to prevent further medical attention delays.

## Introduction and background

Narcolepsy is a persistent sleep disorder that has a particular onset in teenagers and is marked by excessive daytime sleepiness (EDS), which could have a significant impact on the life of the patient and certain psychosocial consequences that make it difficult to excel academically or in workplaces [1]. Social stigma is often a difficulty faced by people suffering from narcolepsy, besides complications in schooling for young adults, disturbed work life, a less pleasant lifestyle, and adverse socioeconomic results are seen. Narcolepsy can be subdivided into type 1 and type 2 (both types have analogous medical profiles except for cataplexy, which is seen in narcolepsy type 1) [2]. It goes on to be a notably underdiagnosed/misdiagnosed situation globally. According to the National Institutes of Health (NIH), an anticipated 150,000 to around 200,000 people in the United States of America are dwelling with narcolepsy. This quantity may be higher because of the wide variety of sufferers who either are no longer trying to find medical aid for the manifestations they face or acquire a wrong initial prognosis at the onset [3]. Cataplexy is a recurrent feature of narcolepsy type 1, which is muscle weakness or semi or total loss of control in reflex to intense emotions, such as sudden bursts of laughter or crying [4].

Cataplexy can injure the person affected as muscle weakness causes the person to slump over or fall. The frequency of cataplexy ranges from many times a day to a few times a year and persists only for a few seconds [5]. Remarkably, at all times, the clinical occurrence of this is attributable to the lack of around 40,000 hypocretin-containing neurons in the lateral hypothalamus with regard to narcolepsy type 1 [6]. The complete stretch of clinical symptoms, which are EDS, sleep paralysis, cataplexy, and hypnagogic hallucinations, is generally absent during the preliminary stages of the disease, which delays the diagnosis during childhood [7]. Sleep paralysis and hypnagogic hallucinations conclude the tetrad of narcolepsy; interrupted sleep at night, impulsive and spontaneous behaviors, and weight gain are common complaints [8]. Narcolepsy can be commonly confused with epilepsy or a psychiatric disorder, for which proper diagnostic history, multiple sleep latency test (MLST), polysomnograms, and even measures of cerebrospinal fluid (CSF) hypocretin are needed for detection [9]. Rapid eye movement (REM) sleep behavior disorder is almost entirely linked with disorders related to neurodegeneration, but it can also be seen in narcolepsy type 1. It is denoted by dream-enacting manners and reduced motor inhibition during REM sleep [10]. The human leukocyte antigen class II (HLA DR2) haplotype is a set of DNA variations that are seen to be correlated to narcolepsy and also implies that narcolepsy could be a result of autoimmune action [11].

Narcolepsy patients are treated in an artistically scientific manner while maintaining the balance between the efficiency of the drug, the amenity of administration, checking for side effects and tolerance to the drug, and then choosing the apt drug for the treatment [12,13]. Currently, narcolepsy has no permanent cure, but prevailing treatments have made lifestyle easier for people suffering from this condition. The treatment can be pharmacological and nonpharmacological [14]. Non-drug methods include short naps of about 20-25 minutes and a stable sleep schedule, which is also composed of enough sleep at night. Caffeine intake in a certain amount can also prove helpful and reduce the use of pharmacological treatment, but this only helps to a certain extent, after which drug intervention is necessary [15]. Sodium oxybate has been proven to be the only helpful drug in the case of cataplexy [16]. Narcolepsy is often misunderstood, and knowledge regarding it is exceedingly less, which leads to delays in diagnosis as it might be confused with laziness or being fake. The feeling of social isolation has also been reported in patients suffering from narcolepsy [17]. Better education, among others, might help those suffering from this condition and also raise awareness about it [18]. This review aims to highlight the four main symptoms of narcolepsy, the advancement in its treatment and management, and the social construct that still follows narcolepsy leading to decreased mental health among its patients.

## Review

Methodology

**Figure 1:**
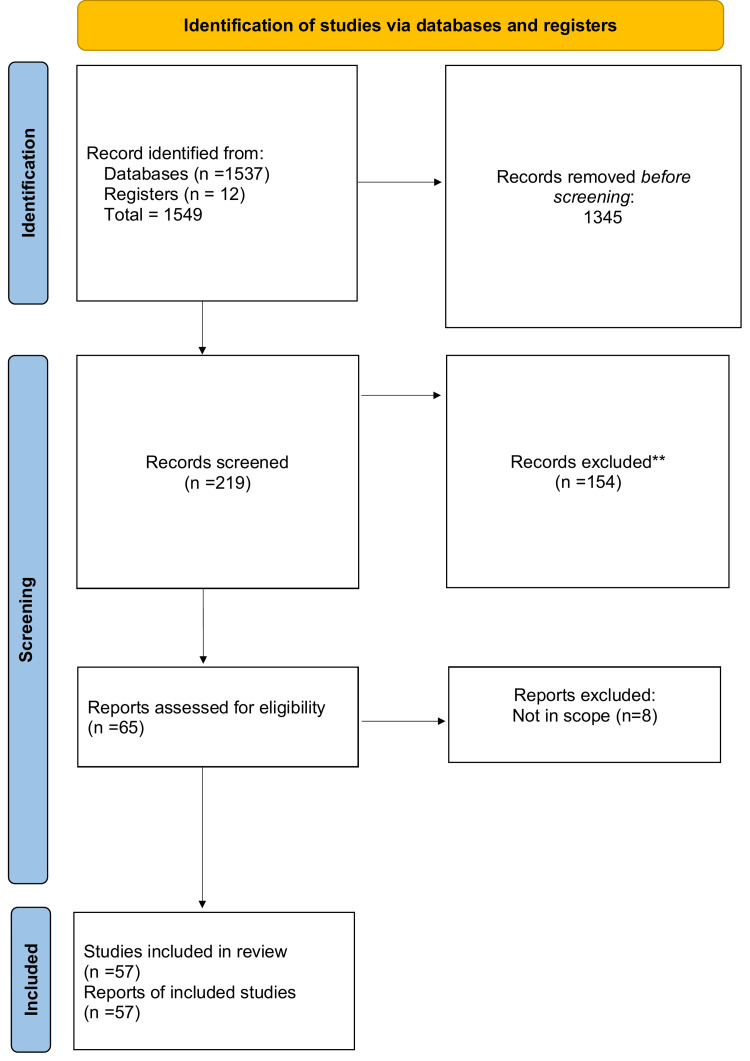
Selection process of articles used in this study Adopted from the Preferred Reported Items for Systematics Reviews and Meta-Analysis (PRISMA)

"The tetrad of narcolepsy" is a systematic review. For this article, we reviewed the PubMed and Google Scholar databases for articles on narcolepsy, its main symptoms, and advances in diagnosis and treatment. We searched for and included around 57 relevant articles and included MeSH terms, such as "narcolepsy," "catalepsy," "symptoms," "diagnosis," and "management." Other keyword combinations included "sleep disorders," "tetrad," and "delayed diagnosis" (Figure 1).

What is narcolepsy?

Narcolepsy is a persistent sleep disorder that has a particular onset in teenagers and is marked by the aid of immoderate daytime sleepiness, which could have excessive effects on the patient [2]. Type 1 narcolepsy is distinguished by cataplexy, which is muscle feebleness caused by intense emotional responses. The decrease or absence of hypocretin (orexin) neurotransmitter is the paramount reason for the cause of type 1 narcolepsy in people who suffer from it [19]. Symptoms, which can be acute or chronic, start to show within a few months to a few years, of which EDS is the first, followed by cataplexy [20]. According to some studies, narcolepsy affects more males than females and children, while some say the contrary. It can affect any age group regardless of gender [21]. The typical tetrad of narcolepsy was given in the year 1956 by Yoss and Daly, which included EDS, hypnagogic hallucinations, sleep paralysis, and cataplexy [22]. Narcolepsy patients might also develop symptoms related to neuropsychiatry, which makes the diagnosis further difficult. Therefore, this should necessarily be kept in mind while diagnosing a narcoleptic patient [23]. Hypertension and the risk of cardiovascular diseases are also prevalent in these patients [24].

EDS

All narcolepsy patients portray symptoms of excessive napping during daytime, which is not voluntary, i.e., “against one’s own will” [22]. Most of the time, EDS comprises a constant sleepy feeling in the background and, a few times, a robust uncontrollable urge to sleep that might occur many times in a day and mostly at unsuitable timings, such as while having a meal. Dull or tedious situations may increase the risk of falling asleep [25]. It is more likely called a “sleep attack.” However, in between these sleep attacks, there is absolute consciousness among individuals, especially while doing a task that keeps them attentive [26]. EDS is also related to diseases, such as Parkinson’s disease, affecting 25-50% of the people suffering from it [27]. Luckily, the drugs already present have been successful in treating and lowering EDS in some patients [28]. Drugs that stimulate the central nervous system (CNS), like modafinil, armodafinil, and wake-promoting compounds, find their application in the treatment [29]. Delays in the recognition of EDS and hence narcolepsy can be prevented by sleep tests, such as the Epworth Sleepiness Scale, thus beginning the appropriate treatment for narcolepsy at the earliest [30]. Another symptom that has quite often been unrecognized is disrupted nighttime sleep (DNS). It does not have a particular set definition, but it refers to the inability to sleep continuously, which may occur following hypnagogic hallucinations in some patients [31]. Only a few percent of patients have all five symptoms, and therefore DNS often goes unrecognized, and more research is needed in this area [32].

Hypnagogic hallucinations and sleep paralysis

The narcoleptic tetrad also comprises sleep paralysis and hypnagogic hallucinations [8]. Hypnagogic hallucination refers to brief, frequently visual, or auditory experience, which may be unpleasant, frightening dreams/nightmares, while transitioning from an awake state to a sleeping state, and about 20-70% of people affected by narcolepsy experience hypnagogic hallucinations and sleep paralysis, known as “REM sleep behavior disorder” [33]. In complete mindfulness, however, the people suffering are unable to move any voluntary muscles even when they are not asleep. This is known as sleep paralysis and happens right before sleeping at night or when getting up in the morning [22]. Hallucinations and sleep paralysis can also cause DNS. DNS is much less diagnosed as compared to EDS and can lead to reduced quality of life [32]. REM sleep behavior sees an increase in the activity of muscles during REM sleep, which may possibly turn out harmful for the patient. REM suppressants can treat hypnagogic hallucinations [34].

Cataplexy

Cataplexy is the foremost symptomatic marker of narcolepsy. It is marked by sudden occurrences of the weakness of muscles in response to strong positive emotions. Except for the diaphragm and eye muscles, all antigravity muscles are affected, causing the person to slump over or fall [35]. Most commonly, the head drops forward, the arms fall to the side, and the unlocking of the knees takes place [36]. The absence or depletion of hypocretin (orexin) neurotransmitters is supposed to be the cause of cataplexy. Cataplectic episodes can be draining for the patient, leaving them partially or completely paralyzed even when awake. These episodes usually persist for a few seconds to a few minutes, varying from person to person, but rarely for hours, known as “status cataplecticus” [37]. An abiding theory in sleep disorders suggests that cataplexy is caused when REM sleep intrudes into wakefulness [38]. A study shows that patients, mainly men, had more common involvement of muscle of the shoulder, falling off the head and jaw, and such episodes would occur more than once a month [39].

Pathophysiology

Orexins play an important role in the physiology of sleep. Regions related to sleep-wake states, motivation, circadian phase, etc., project their input signals toward orexin neurons, which further direct the output signals toward areas of the brain that maintain wakefulness and suppress and regulate REM sleep and non-REM sleep [40]. Orexins, also known as hypocretins, have been known to regulate serotonin neurons in the dorsal raphe nucleus [41], which is released during the waking state in the highest proportion in cortical and subcortical regions, as seen in microdialysis experiments [42]. It also activates nor-adrenergic and histamine neurons in the lateral hypothalamus, making its physiological role in sleep a bit complex [43]. While low levels of orexin can be one of the causes of type 1 narcolepsy, the cause of type 2 narcolepsy, symptoms of which are less severe, is still unknown [40].

Research has been going on about narcolepsy, which has been studied for nearly 150 years, but the underlying cause became comprehensible only in the past 20 years. Two types of orexins, namely, A (hypocretin 1) and B (hypocretin 2), were discovered in 1998. Soon, stunted levels of orexin in CSF because of partial or complete loss of orexin neurons were found to be the cause of narcolepsy [44]. REM sleep behavior may also be linked with orexin loss; with recent advances, it has started becoming more lucid. REM sleep behaviors, such as muscular atonicity and dreaming, are differentially affected by hypocretin, besides which REM sleep might also be inhibited by it [45]. In 1998, research conducted on dogs and mice by Stanford researchers and Chemelli et al., respectively, identified hypocretins as the key sleep-regulating neurotransmitter [46]. Following this, the role of hypocretin was analyzed in humans, and it was found that those with type 1 narcolepsy had stunted levels of hypocretin-1. By contrast, gene mutation was identified as a less significant cause of the condition [46]. Numerous regions and nuclei in the brain that regulate sleep receive and send projections to hypocretin neurons. This shows that the hypocretin system corresponds to histaminergic, cholinergic, serotonergic, and GABAergic systems. In this condition, the loss of hypocretin transmission is in the same proportion as the impairment of insulin in type 1 diabetes [47].

The cause of the destruction of orexin neurons is yet to be known, but there is quite some evidence that proves that type 1 narcolepsy is an autoimmune disorder mediated by CD4+ T cells [36]. Narcolepsy is very closely correlated to the human leucocyte antigen (HLA) DQB1*0602 allele, for which most of the patients are positive. However, this is not a definitive feature of narcolepsy, yet it escalates the risk of developing the condition by 200 times [37]. Altogether, it is believed that in individuals with a positive DQB1*0602 allele, an abnormal humoral and cellular immunological response was set off by an environmental factor, such as a streptococcal infection [38]. This immunological response, which is thought to be mediated by CD4+ and CD8+ T cells, destroys the orexin neurons [39].

Diagnosis

Narcolepsy is a lifelong disease in which many years could pass by without a diagnosis and is also quite often misdiagnosed [48]. Narcolepsy can be commonly confused with epilepsy or a psychiatric disorder, for which proper diagnostic history, MLST, polysomnograms, actigraphy, and even measures of CSF hypocretin are needed for detection [9]. Diagnosis is made by looking into the history of symptoms of the patient. EDS is the most common symptom wherein sleep attacks are seen. Cataplexy is another major symptom [3]. Polysomnograms may help assess obstructive sleep apnea and monitor electromyogram, electrocardiogram, and eye movements. To detect sleep-wake patterns and movements during sleep, a device known as an actigraph is placed on the patient’s wrist [27]. Actigraphy has its role in sleep medicine as a diagnostic tool [49]. MSLT is used when no sleep disorder is recognized and enough sleep is recorded on a polysomnogram. It is based on the assumption that sleep latency decreases if normal sleeping time increases. Various parameters have been used to examine the reliability of MSLT like test-retest and scoring. The American Academy of Sleep Medicine established the standard for performing MSLT [50]. It measures the proneness of falling asleep under controlled situations. Every two hours a day, four to five naps are recorded, after which the mean sleep latency is used. MSLT varies under certain circumstances and may not be completely reliable sometimes [51].

Treatment and management

The treatment of narcolepsy, just like any other chronic condition, is more symptomatic instead of curative management, which is pharmacological and non-pharmacological [50]. Non-drug methods include short naps of about 20-25 minutes and a stable sleep schedule, which is also composed of enough sleep at night. Caffeine intake in a certain amount can also prove helpful and reduce the use of pharmacological treatment. However, this only helps to a certain extent, after which drug intervention is necessary [15]. Children with narcolepsy need special attention and a separate schedule designed for them. People with this condition work better in environments that do not require much sitting hours as that would worsen the tendency to sleep. Family and peer understanding and cooperation play a major part in helping them get through the condition as there is no ultimate cure. Pharmacological intervention is certainly needed. Drugs, such as modafinil, armodafinil, methylphenidate, pitolisant, solriamfetol, and amphetamines, have been proven to be useful in treating narcolepsy but have certain side effects and cannot be given to a patient with a history of cardiovascular diseases. Sodium oxybate, a sodium salt belonging to gamma hydroxybutyrate, has proven to be very useful in the case of cataplexy [50] (Table 1).

**Table 1:** Description of drugs with evidence of safety and efficacy EDS: excessive daytime sleepiness, ESS: Epworth Sleepiness Scale References: [51,56,57]

Sr. No.	Drugs	Effects	Evidence
1.	Modafinil	A strong dosage helps in EDS and thus improves the quality of life.	In a clinical trial conducted to see the efficacy of modafinil in patients with narcolepsy, unsatisfied with the previous treatment, 151 patients were enrolled in an open-label, six-week clinical trial, received 200-400 mg modafinil and were evaluated by ESS. Modafinil turned out to be an effective treatment for EDS in narcolepsy [52].
2.	Armodafinil	Conditional, helps in EDS and cataplexy.	A 12-week randomized control trial where 196 patients of EDS associated with narcolepsy were subjected to 150 mg and 250 mg armodafinil and placebo. The efficacy was evaluated based on the maintenance of wakefulness test (MWT). Armodafinil seemed to increase sleep latency significantly compared to placebo in patients with narcolepsy. It also improved memory and attention [53].
3.	Pitolisant	Strong dosage, also helps in EDS.	An eight-week randomized control trial known as Harmony 1, conducted between May 2009 and June 2010, included 110 patients who had EDS, out of which 32 were randomly assigned to pitolisant treatment. The findings concluded that this drug is effective for EDS and could be an option for the treatment of narcolepsy. The main purpose of the trial was to point out the difference between pitolisant, placebo, and modafinil with respect to ESS results [54,55].
4.	Sodium oxybate	Strongly recommended for cataplexy	A 12-week randomized control, open-label trial included treatment of patients of narcolepsy with cataplexy, previously on anticataleptics, who were given lower sodium oxybate (LXB). When given alone, LXB reduced the episodes of cataplexy. When asked to switch from anticataleptics to LXB, cataplexy happened more often in very few patients but later reduced, making it evident that switching to LXB would prove beneficial for cataplexy without it becoming worse [56].
5.	Solriamfetol	It is a reuptake inhibitor of epinephrine and norepinephrine for the treatment of EDS.	A randomized trial of 12 weeks was conducted on patients with narcolepsy, which included 236 patients assigned to treatment with solriamfetol 300 mg. The end results concluded that this drug could treat impaired wakefulness and EDS in narcolepsy [57].

Even with all the diagnoses and drug interventions, there is no certain treatment for narcolepsy, and more research in this field is still needed.

## Conclusions

Narcolepsy is a chronic condition that, despite many advances in its diagnosis, treatment, and management, has still not found a permanent cure and continues to be misdiagnosed or underdiagnosed due to a lack of knowledge and education about the same. The famous tetrad of symptoms has been the main highlight of this review article. People suffering from it often struggle with anxiety and depression and are many a time misunderstood as lazy. The constant feeling of being tired and wanting to sleep has led to social anxiety and isolation among many. Narcolepsy still needs to be studied in detail in adults and children, and education among those who do not suffer from it is also very important so as to relieve the patients from any social construct.
